# 

**Published:** 1914-03-21

**Authors:** 


### THE COUPON.

#### THE DOCTORS' WIVES ASSOCIATION.

I hereby certify that I am a doctor's wife, and I beg to send you my name and address as one who wishes to become a member of the proposed Doctors' Wives Association. I hereby agree to qualify as a member by paying the subscription of 7s. 6d. per annum on or before May 1, 1914, such subscription entitling me each year to a free copy of The Hospital newspaper delivered weekly, with the Doctors' Wives section, and to include my annual subscription to the Association.

This agreement undertaking to subscribe on my part is subject to at least 1,000 doctors' wives conditionally joining the proposed Association by signing and returning to "Co-operation" as below by April 18 next a similar coupon to that to which I hereby attach my signature.

(Signed) ..........................................

(Wife of) * ..........................................

(Address) ..........................................

..........................................

(Date) ..........................................

*Note.*—When filled in and signed cut out this coupon, place it in an envelope and address and post it to:

Co-Operation,
c/o The Scientific Press,
28 & 29 Southampton Street, Strand,
London, W.C.

* Here insert full name and qualifications.

RATES OF SUBSCRIPTION: Twelve months, 6/6; Six months, 4/-; Three months, 2/-, post free to any address in the United Kingdom. Abroad, Twelve months, 8/8; Six months, 5/-; Three months, 2/6, post free.—Address The Subscription Dept., "THE HOSPITAL," The Hospital Building, 28/29 Southampton Street, Strand, London, W.C.

